# High self-reported non-adherence to antiretroviral therapy amongst adolescents living with HIV in Malawi: barriers and associated factors

**DOI:** 10.7448/IAS.20.1.21437

**Published:** 2017-03-30

**Authors:** Maria H Kim, Alick C Mazenga, Xiaoying Yu, Saeed Ahmed, Mary E Paul, Peter N Kazembe, Elaine J Abrams

**Affiliations:** ^a^ Baylor College of Medicine International Paediatric AIDS Initiative, Texas Children’s Hospital, Houston, TX, USA; ^b^ Baylor College of Medicine- Abbott Fund Children’s Clinical Centre of Excellence, Lilongwe, Malawi; ^c^ Department of Medicine, Baylor College of Medicine, Houston, TX, USA; ^d^ Design and Analysis Core, Baylor-UT Houston Center for AIDS Research, Houston, TX, USA; ^e^ ICAP, Mailman School of Public Health and College of Physicians & Surgeons, Columbia University, New York, NY, USA

**Keywords:** Antiretroviral therapy, HIV, adherence, adolescents, sub-Saharan Africa, alcohol, violence, self-efficacy

## Abstract

**Introduction**: Globally adolescents and young adults account for more than 40% of new HIV infections, and HIV-related deaths amongst adolescents increased by 50% from 2005 to 2012. Adherence to antiretroviral therapy (ART) is critical to control viral replication and preserve health; however, there is a paucity of research on adherence amongst the growing population of adolescents living with HIV/AIDS (ALHIV) in Southern Africa. We examined levels of self-reported ART adherence, barriers to adherence, and factors associated with non-adherence amongst ALHIV in Malawi.

**Methods**: Cross-sectional study of 519 ALHIV (12–18 years) attending two large HIV clinics in central and south-eastern Malawi. Participants self-reported missed doses (past week/month), barriers to adherence, and completed questionnaires on past traumatic events/stressors, disclosure, depression, substance use, treatment self-efficacy, and social support. Biomedical data were retrieved from existing medical records. Multivariate logistic regression was performed to identify factors independently associated with self-reported ART adherence (7 day recall).

**Results**: The mean age of participants (SD) was 14.5 (2) years and 290 (56%) were female. Of the 519 participants, 153 (30%) reported having missed ART doses within the past week, and 234 (45%) in the past month. Commonly reported barriers to adherence included forgetting (39%), travel from home (14%), busy with other things (11%), feeling depressed/overwhelmed (6%), feeling stigmatized by people outside (5%) and within the home (3%). Factors found to be independently associated with missing a dose in the past week were drinking alcohol in the past month (OR 4.96, 95% CI [1.41–17.4]), missed clinic appointment in the past 6 months (OR 2.23, 95% CI [1.43–3.49]), witnessed or experienced violence in the home (OR 1.86, 95% CI [1.08–3.21]), and poor treatment self-efficacy (OR 1.55 95% CI [1.02–2.34]). Sex and age were not associated with adherence.

**Conclusions**: In our study, nearly half of all ALHIV reported non-adherence to ART in the past month. Violence in the home or alcohol use in the past year as well as poor treatment self-efficacy were associated with worse adherence. Sub-optimal adherence is a major issue for ALHIV and compromise treatment outcomes. Programmes specifically tailored to address those challenges most pertinent to ALHIV may help improve adherence to ART.

## Introduction

Globally there are 2.1 million adolescents living with HIV (ALHIV) [1]. The majority, 1.7 million live in sub-Saharan Africa (SSA) [1]. HIV is the second leading cause of adolescent morbidity and mortality worldwide and the leading cause in Africa [1]. Furthermore, in the same period that AIDS-related deaths decreased by 30% globally, AIDS-related deaths amongst adolescents aged 10–19 years old grew by 50% [2,3].

Effective antiretroviral therapy (ART) results in virologic suppression, immune reconstitution and decreased morbidity. However, the relationship between ART and virologic suppression is mediated by excellent adherence [4,5]. Poor adherence can lead to viral rebound, disease progression, and drug resistance which can compromise patient clinical outcomes and increase the risk of transmitting resistant strains of HIV to others when adolescents become sexually active [4,6–8]. Unfortunately, adherence to ART among children and adolescents is suboptimal [4,5,9,10]. A recent systematic review and meta-analysis [4] that included over 50 studies globally found that only 62.3% of the adolescent and young adult (AYA) population were classified as adherent to therapy assessed by self-report and plasma viral HIV RNA load (VL) levels (adherence at least 85% on self-report or undetectable blood plasma virus levels).

Adolescence is a period of vulnerability for well-documented biological, developmental, and behavioural reasons. It is characterized by less parental support and supervision, decreased inhibition, increased risk-taking, and immature judgement [4,11–13]. Adolescents living with HIV/AIDS (ALHIV) have the additional rigorous demands of a chronic disease that require life-long daily antiretroviral therapy (ART) [4,5]. In combination, this makes ALHIV particularly vulnerable to poor ART adherence [4,5,9,10].

Prior studies have reported on a variety of barriers to and factors associated with suboptimal ART adherence amongst ALHIV [14]. These factors may act simultaneously and their effects can vary depending on the particular environmental/cultural setting [4]. Associations have been found between socio-demographic factors such as age [10,15–17], gender [18], structural and economic factors like distance from the treatment centre [19], past traumatic events/stressors like loss of a family member [20], violence in the home [21], or bullying; behavioural/psychosocial factors such as treatment self-efficacy (one’s sense of being able to adhere to ART as prescribed), forgetting to take ART [22,23], alcohol and drug use [24], and HIV disclosure to another person [25], and bio-clinical or treatment-related factors [10,22].

HIV is the leading cause of adolescent morbidity and mortality in Africa [1]. Although southern Africa is home to the countries with the highest HIV prevalence, there is limited data on adherence amongst ALHIV from this region outside of South Africa. Further there are few studies examining the impact of important adherence modifying factors such as violence and adherence self-efficacy amongst ALHIV in southern Africa. The lack of evidence hinders the development of interventions to address adherence challenges and improve treatment outcomes. Malawi is a land-locked country in southern Africa and ranks as one of the poorest countries in the world by annual GDP per capita [26]. The HIV prevalence is 10.8%, and there are an estimated 83,000 ALHIV living in Malawi, making Malawi one of the top 10 countries in terms of numbers of ALHIV [2,27]. The main objective of this study was to describe self-reported adherence, barriers to adherence, and examine factors associated with suboptimal adherence amongst ALHIV in Malawi.

## Methods

### Study design, population and setting

There were 562 participants, recruited to a cross-sectional study (January–August 2012) of perinatally HIV-infected 12–18 year olds in Malawi. Study procedures for the larger study are fully described elsewhere [28,29]. In brief, we recruited a convenience sample of adolescents enrolled at the Baylor College of Medicine Children’s Clinical Center of Excellence (COE) and Zomba ART Clinic. The Baylor COE and Zomba Clinic are the largest pediatric HIV clinics in central and south-eastern Malawi, respectively. Both clinics offer free outpatient care in partnership with the Malawi Ministry of Health (MOH). At the time of the study, the first-line regimen for adolescents was a fixed-dose combination of zidovudine, lamivudine and nevirapine given twice daily; second-line regimen was abacavir, lamivudine and lopinavir/ritonavir. Consents/assents were obtained from caregivers/adolescents. Adolescents completed a battery of questionnaires in Chichewa (the local language). The surveys were administered anonymously to reduce measurement bias and enhance the validity of self-report data. After obtaining written consent, study staff gave participants the self-administered surveys. As part of the instructions, participants were assured that their names and other identifying information would not be written anywhere on the surveys. Of the 562 participants in the larger study, 519 were on ART and included in the present analysis. Participants that were new to ART, those established on ART, as well as those on second-line regimens were all included.

### Measures

#### ART adherence and HIV treatment self-efficacy

Questions to measure adherence and adherence self-efficacy were adapted from those used in prior studies [30,31]. Specifically, ART adherence was the primary outcome and was measured by self-reported answers to the following questions: “Have you ever missed taking your medicine in the past 7 days” and “Have you ever missed taking your medicine in the past 30 days”? Participants were also asked “Did you miss any clinic appointments in the past 6 months?”

Self-efficacy for antiretroviral adherence is one’s sense of being able to adhere to ART as prescribed. We measured treatment self-efficacy with three questions adapted from prior studies [30,31]. The three questions were: (1) how sure are you that you will be able to take all of your medication as directed? (2) how sure are you that your medication will have a positive effect on your health, and (3) how sure are you that your medicines will help you to live a long and healthy life. Participants responded on a 4-point Likert-type scale (not sure, somewhat sure, very sure, or extremely sure). Responses were combined into a composite dichotomous variable – not extremely sure, and extremely sure. Reliability was acceptable with Cronbach’s alpha 0.89.

#### Barriers to medication adherence

Participants completed a self-administered 19-item checklist of barriers to adherence to ART modified from the P1042S Child/Adolescent Questionnaire [23]. Adjustments were made to improve contextual relevance and understanding. For example, questions on food insecurity and transportation problems were added. Barriers were classified as logistical, regimen related, adolescent related, knowledge/attitudes, stigma, and emotional. The respondents were asked how often each barrier had contributed to missed medications in the past month. They were asked to rate the frequency of the barrier by ticking one of four choices on a Likert-type scale (never, rarely, sometimes, often).

#### Depression

Depression was measured using the self-administered Becks Depression Inventory (BDI) and the clinician assessed Children’s Depression Rating Scale-Revised (CDRS-R). Both measures have been validated in diverse settings and cultures [32–37]. A locally validated and culturally adapted Chichewa version of the BDI was used [28,29]. The Cronbach’s alpha for the BDI was 0.80 [28,29]. The larger study encompassed psychometric evaluations of the depression screening instruments and these findings have been reported fully elsewhere [28,29]. In summary, the BDI-II is a 21-items self-rated depression screening instrument that evaluates both the presence and severity of depressive symptoms in those aged 13 years and over [33]. CDRS-R is clinician assessed and is the most widely used rating tool for the assessment of depressive symptoms in children and adolescents, particularly in international research trials [34–37]. A specialist Mental Health Clinician trained and supervised the clinician interviewers who performed the clinician administered CDRS-R.

#### Socio-demographic and behavioural factors

Data on socio-demographic and behavioural variables thought to be potentially associated with adherence were collected using a self-administered survey. The variables are listed in Table 1 and include data on socio-demographics (gender, age, grade in school, location of the home, travel time to the clinic, primary caregiver type) and structural/economic factors such as income level; past traumatic events/stressors such as deaths in the family, history of sexual or domestic violence, victim of teasing/bullying; behavioural factors/social support such as alcohol use and disclosure of HIV status. For the question “to what extent do your friends and family members help you to remember to take your medication”, there were three potential answers ((a.) Not at all, (b.) A little, (c.) A lot). We dichotomized the response by combining “a little” and “a lot” to facilitate interpretation.

**Table 1.** Characteristics of study participants, self-reported adherence (7 day recall)

Variable	Total *N* (%)	Not Adherent *n* (%)	Adherent *n* (%)	*p*-value (Chi-sq)
Age, median (IQR), years^a^	14 (13, 16)	14 (13, 16)	14 (13, 16)	0.94^a^
Gender				
Female	290 (55.9)	88 (57.5)	202 (55.2)	0.63
Male	229 (44.1)	65 (42.5)	164 (44.8)	
School Grade				
Not in school or Junior Primary School	161 (31)	51 (33.3)	110 (30.1)	0.32
Senior Primary School	212 (40.8)	66 (43.1)	146 (39.9)	
Secondary School or Tertiary	146 (28.1)	36 (23.5)	110 (30.1)	
Family’s estimated combined income				
Less than 14,000 MK per month	161 (32.5)	48 (33.1)	113 (32.3)	0.54
14,000–49,999 MK per month	105 (21.2)	26 (17.9)	79 (22.6)	
More than 50,000 per month	67 (13.5)	18 (12.4)	49 (14)	
I do not know	162 (32.7)	53 (36.6)	109 (31.1)	
Location of home				
In the city	337 (64.9)	102 (66.7)	235 (64.2)	0.10
Just outside the city	87 (16.8)	18 (11.8)	69 (18.9)	
Rural area	95 (18.3)	33 (21.6)	62 (16.9)	
Time it takes to get to the clinic from home				
0–30 min	66 (12.7)	19 (12.5)	47 (12.8)	0.99
31–60 min	209 (40.3)	61 (40.1)	148 (40.4)	
>60 min	243 (46.9)	72 (47.4)	171 (46.7)	
Primary caregiver type				
Father/Mother	167 (32.2)	47 (30.7)	120 (32.8)	0.053
Both parents	104 (20)	21 (13.7)	83 (22.7)	
Uncle/Aunt	124 (23.9)	40 (26.1)	84 (23)	
Other/Grandparent	124 (23.9)	45 (29.4)	79 (21.6)	
Experience of family/household deaths				
Nobody in my family has died	138 (26.6)	31 (20.3)	107 (29.2)	0.03
One or more people have died	381 (73.4)	122 (79.7)	259 (70.8)	
Witnessed household violence or experienced violence (forced sex or physical violence) in the past year				
No	443 (85.4)	117 (76.5)	326 (89.1)	0.0002
Yes	76 (14.6)	36 (23.5)	40 (10.9)	
Missed clinic appointment in the past 6 months				
Yes	128 (25.1)	56 (37.6)	72 (19.9)	<0.0001
No	382 (74.9)	93 (62.4)	289 (80.1)	
Use of alcohol in the past 30 days				
Never	506 (97.5)	144 (94.1)	362 (98.9)	0.003^b^
Once a month or more	13 (2.5)	9 (5.9)	4 (1.1)	
Years on ART-categorical				
Six years or less	403 (78.4)	112 (73.7)	291 (80.4)	0.09
More than six years	111 (21.6)	40 (26.3)	71 (19.6)	
Efavirenz-based ART regimen				
Yes	36 (6.9)	8 (5.2)	28 (7.7)	0.32
No	483 (93.1)	145 (94.8)	338 (92.3)	
Second line ART regimen				
No	450 (86.7)	134 (87.6)	316 (86.3)	0.70
Yes	69 (13.3)	19 (12.4)	50 (13.7)	
Initial WHO stage				
WHO stage 1–2	129 (25)	39 (25.8)	90 (24.7)	0.78
WHO Stage 3–4	387 (75)	112 (74.2)	275 (75.3)	
Most recent CD4 count, median (IQR)^a^	477 (293, 678)	507 (300, 729)	460 (288, 657)	0.23^a^
HIV immunological classification (based on CD4)				
None or not significant	217 (46.3)	69 (51.1)	148 (44.3)	0.23
Mild	97 (20.7)	23 (17)	74 (22.2)	
Advanced	65 (13.9)	22 (16.3)	43 (12.9)	
Severe	90 (19.2)	21 (15.6)	69 (20.7)	
BMI for age z-score, mean (SD)^c^	−0.9 (1.2)	−0.8 (1.3)	−0.9 (1.2)	0.15^c^
Height for age z-score, mean (SD)^c^	−1.8 (1.2)	−1.8 (1.2)	−1.8 (1.2)	0.65^c^

^a^Median and IQR, and Wilcoxon rank sum test *p*-value was reported. ^b^Fisher’s exact test. ^c^Mean and SD, and two-sample t-test *p*-value was reported.

#### Bio-clinical parameters

Bio-clinical variables were extracted from the existing patient electronic medical record and included: ART start date and regimen; history of hospital admissions, TB, and malnutrition; initial and most recent WHO stage, initial and most recent CD4/immunological classification, BMI-for-age z-score, and height-for-age z-score.

### Ethical considerations

The National Health Sciences Research Committee (NHSRC) of Ministry of Health in Malawi and the Baylor College of Medicine Institutional Review Board in the USA approved the study protocol. We obtained written informed consents from the guardians and written assents from the study participants. All participants determined to have moderate-to-severe depression or suicidal ideation were referred to onsite mental health professionals.

### Statistical analysis

Descriptive statistics, such as mean, standard deviation (SD) for continuous variables, and frequency and proportion for categorical variables, were calculated. Chi-square test or two-sample t-test was used to explore the association between potential factors and ART adherence. For CDRS-R score, we defined depression by having a score equal to or higher than 55 [28]. Categories with sparse cells or similar outcome values were collapsed. For correlated variables, which measured similar features, we either combined the variables or selected the most important variable for further analysis. Malnutrition was categorized according to National Center for Health Statistics reference standards. Z-scores for BMI and height-for-age were calculated using WHO growth standards. Adherence was evaluated as a dichotomous variable. Non-adherence was measured as self-reporting having missed a dose of ART in the past 7 days (7 day – recall).

In model building, initial univariate analysis was performed and all variables with *p*-value less than 0.25 were included in a second round of screening by adjusting for age and sex. Eleven candidate variables including school grade, primary caregiver type, experience of familial/household deaths, witnessed or experienced household violence in the past year, experience of being bullied for taking medications, hospital admission in the past year, depression, missed clinic appointment in the past 6 months, self-efficacy, use of alcohol in the past month, and years on ART were entered into the multivariate regressions. We included age and sex in the models regardless of the statistical significance, and performed backwards selection on other variables with significance *p* ≤ 0.05. Only variables with a *p*-value <0.05 were retained in the final model (Table 4). The scale of continuous variables such as, total BDI score and time on ART was checked by quartiles and clinically relevant cut offs. For duration of ART treatment, we split subjects based on time period of ART treatment into 4 groups. The cut off based on quartiles was 2, 3.5, and 6 years. We found that subjects with ART duration >6 years had significantly increased risk for non-adherence, while groups with shorter ART duration had similar risk, therefore we decided on the cut off of ≤6 vs. >6 years in the modelling. Cut offs for the BDI scores were as follows: 0–13: minimal depression, 14–19: mild depression, 20–28: moderate depression, 29–63: severe depression. We examined the risk of non-adherence among these groups and found a roughly linear trend – increasing risk of non-adherence with increasing BDI scores. Therefore, we included the total BDI score as a linear scale in the model

The final model with selected main effects was checked by model diagnostic techniques, such as, residual analysis and influence statistics. The overall fit was assessed by Hosmer and Lemeshow Goodness-of-Fit. The odds ratio (OR) and 95% confidence interval (CI) were reported to evaluate the associations between the covariates and outcome adjusting for other covariates.

Self-reported barriers to adherence were classified under domains (Table 4). A binary variable (Yes, No) was created to indicate any barrier in each domain. Items with response “rarely”, “sometimes” or “often” within a given domain/barrier were classified as Yes. We compared the proportions of barriers for each domain/barrier by Fishers exact test.

A *p*-value <0.05 was considered statistically significant and SAS software version 9.4 (SAS Institute, Cary, N.C.) was used for all analyses.

## Results

The mean age of participants was 14.5 (SD 2) years and 290 (56%) were female (Table 1). There was no statistically significant relationship between age or gender and adherence. Of the 519 participants, 153 (30%) reported having missed one or more ART doses within the past week, and 234 (45%) in the past month. There were 451 (87%) participants on 1st line ART regimens, and 69 (13.3%) on 2nd line regimens. On average, participants had been on ART for 3.74 years (SD: 2.23).

### Univariate analysis of associations between self-reported non-adherence and other variables

After adjusting for age and gender (Table 2), variables associated with non-adherence included negative past life experiences in the past year (experiencing household deaths and witnessed household violence or experienced violence), being bullied for taking their medicines, hospital admissions in the past year, missed clinic appointment in the past 6 months, depression (as measured by the CDRS-R, AOR 1.7, 95% CI [1.08–2.70],*p* = 0.02), poor treatment self-efficacy and alcohol use in the past 30 days.

**Table 2.** Factors associated with self-reported non-adherence (7 day recall) unadjusted and adjusted for age and sex

Variable	Unadjusted OR (95% CI)	*p*-value	Adjusted OR (95% CI)	*p*-value
**Socio-demographic factors**				
School Grade				
Not in school or Junior Primary School	1.42 [0.86–2.34]	0.32	1.73 [0.94–3.16]	0.16
Senior Primary School	1.38 [0.86–2.22]		1.57 [0.93–2.64]	
Secondary School or Tertiary	Reference		Reference	
Family’s estimated combined income				
Less than 14,000 MK per month	0.87 [0.55–1.40]	0.54	0.88 [0.55–1.40]	0.53
14,000–49,999 MK per month	0.68 [0.39–1.17]		0.67 [0.39–1.17]	
More than 50,000 per month	0.76 [0.40–1.42]		0.76 [0.40–1.43]	
I do not know	Reference		Reference	
Location of home				
In the city	0.82 [0.50–1.32]	0.10	0.81 [0.50–1.31]	0.09
Just outside the city	0.49 [0.25–0.96]		0.48 [0.24–0.94]	
Rural area	Reference		Reference	
Time it takes to get to the clinic from home				
0–30 min	0.96 [0.53–1.75]	0.99	0.96 [0.53–1.75]	0.99
31–60 min	0.98 [0.65–1.47]		0.97 [0.65–1.46]	
>60 min	Reference		Reference	
Primary caregiver type				
Father/Mother	0.69 [0.42–1.13]	0.06	0.69 [0.42–1.14]	0.06
Both parents	0.44 [0.24–0.81]		0.44 [0.24–0.81]	
Uncle/Aunt	0.84 [0.49–1.41]		0.85 [0.50–1.44]	
Other/Grandparent	Reference		Reference	
**Past traumatic events/stressors**				
Maternal death or employment status				
Not working	0.97 [0.59–1.59]	0.24	0.96 [0.59–1.59]	0.24
Self-employed	0.6 [0.35–1.02]		0.59 [0.34–1.02]	
Employed by someone else	0.72 [0.39–1.33]		0.72 [0.39–1.34]	
Died	Reference		Reference	
Change in caregiver				
No change in caregiver	0.82 [0.53–1.26]	0.36	0.81 [0.53–1.26]	0.35
Caregiver has changed once or more	Reference		Reference	
Experience of family/household deaths				
Nobody in my family has died	0.62 [0.39–0.97]	0.04	0.61 [0.39–0.96]	0.03
One or more people have died	Reference		Reference	
Failed school term/class				
No	0.8 [0.54–1.17]	0.24	0.79 [0.54–1.16]	0.23
Yes	Reference		Reference	
Witnessed household violence or experienced violence (forced sex or physical violence) in the past year				
No	0.4 [0.24–0.66]	0.0003	0.39 [0.24–0.65]	0.0003
Yes	Reference		Reference	
Experience of being bullied for one’s physical appearance				
No	0.81 [0.52–1.24]	0.33	0.81 [0.52–1.26]	0.35
Yes	Reference		Reference	
Experience of being bullied for taking medicines				
No	0.54 [0.31–0.93]	0.03	0.53 [0.30–0.93]	0.03
Yes	Reference		Reference	
Hospital admissions in the past year				
No	0.52 [0.29–0.95]	0.03	0.52 [0.29–0.95]	0.03
Yes	Reference		Reference	
**Behavioural factors/social support**				
Depression Assessment				
BDI score, mean (SD, *N*)	1.02 [1.00–1.05]	0.05	1.02 [1.00–1.05]	0.06
Depression by CDRS-R, yes	1.71 [1.08–2.70]	0.02	1.7 [1.08–2.70]	0.02
Depression by CDRS-R, no	Reference		Reference	
Missed clinic appointment in the past 6 months				
Yes	2.42 [1.59–3.68]	<0.001	2.47 [1.61–3.77]	<0.001
No	Reference		Reference	
How much has your physical or emotional health interfered with your normal social activities in the past month				
Not at all	0.77 [0.44–1.34]	0.28	0.77 [0.44–1.35]	0.28
A little	1.09 [0.56–2.10]		1.1 [0.57–2.13]	
Sometimes	1.25 [0.61–2.55]		1.26 [0.62–2.57]	
A lot	Reference		Reference	
Self-efficacy regarding taking ART (composite of three self-efficacy questions)				
Not extremely sure	1.47 [1.00–2.17]	0.05	1.48 [1.00–2.19]	0.05
Extremely sure	Reference		Reference	
Experience of being in a romantic relationship that did not involve sex				
Never had a boyfriend/girlfriend	0.76 [0.45–1.28]	0.30	0.75 [0.42–1.32]	0.32
Yes, in the past or current	Reference		Reference	
Satisfaction with the way I look (physical appearance)				
I am very happy with the way I look	0.97 [0.60–1.59]	0.92	0.99 [0.60–1.62]	0.97
I am somewhat/not at all satisfied with the way I look	Reference		Reference	
Use of alcohol in the past 30 days				
Never	0.18 [0.05–0.58]	0.004	0.18 [0.05–0.59]	0.005
Once a month or more	Reference		Reference	
To what extent do your friends and family members help you to remember to take your medication?				
Not at all	0.65 [0.35–1.22]	0.18	0.64 [0.34–1.20]	0.16
A lot or a little	Reference		Reference	
HIV disclosure status^a^				
Disclosed and have shared with someone	1.22 [0.71–2.10]	0.78	1.23 [0.68–2.21]	0.79
Disclosed, have not shared with anyone	1.17 [0.66–2.08]		1.18 [0.65–2.14]	
Not disclosed	Reference		Reference	
Age at disclosure	1.04 [0.92–1.19]	0.52	1.06 [0.92–1.23]	0.44
**Bio-clinical parameters**				
Years on ART	1.04 [0.96–1.13]	0.36	1.04 [0.96–1.14]	0.35
Years on ART-categorical				
Six years or less	0.68 [0.44–1.07]	0.09	0.68 [0.43–1.06]	0.09
More than six years	Reference		Reference	
Efavirenz based ART regimen				
Yes	0.67 [0.30–1.50]	0.33	0.67 [0.30–1.50]	0.33
No	Reference		Reference	
Second line ART regimen				
No	1.12 [0.63–1.96]	0.71	1.11 [0.62–1.97]	0.73
Yes	Reference		Reference	
History of tuberculosis treatment				
No	0.93 [0.63–1.35]	0.69	0.92 [0.63–1.35]	0.68
Yes	Reference		Reference	
Initial WHO stage				
WHO stage 1–2	1.06 [0.69–1.64]	0.78	1.07 [0.69–1.66]	0.76
WHO Stage 3–4	Reference		Reference	
Most recent CD4 count	1 [1.00–1.00]	0.25	1 [1.00–1.00]	0.22
HIV immunological classification (based on CD4)				
None or not significant	1.53 [0.87–2.70]	0.23	1.56 [0.88–2.75]	0.22
Mild	1.02 [0.52–2.01]		1.03 [0.52–2.02]	
Advanced	1.68 [0.83–3.42]		1.69 [0.83–3.43]	
Severe	Reference		Reference	
BMI for age z-score	1.13 [0.96–1.32]	0.15	1.13 [0.95–1.33]	0.17
Height for age z-score	1.04 [0.88–1.22]	0.65	1.03 [0.87–1.22]	0.74

^a^Disclosed and have shared with someone: The adolescent knows their HIV status (has been disclosed to) and the adolescent has also shared her/his HIV status with someone else. Disclosed and have not shared with someone: The adolescent knows their HIV status (has been disclosed to) but the adolescent did not share her/his HIV status with someone else. Not disclosed: The adolescent is not aware of their HIV status. OR (odds ratio); SD (standard deviation);WHO (World Health Organization); BDI (Becks Depression Inventory); CDRS-R (Children’s Depression Rating Scale- Revised); ART (antiretroviral therapy); BMI (body mass index).

### Multivariate regression model predicting self-reported non-adherence

In multivariate analysis (Table 3), the variables significantly associated with non-adherence were missing a clinic appointment in the past 6 months, worse treatment self-efficacy (OR 1.55, 95%CI [1.02–2.34]), witnessed or experienced household violence in the past year (OR 1.86, 95%CI [1.08–3.21]), and alcohol use in the past 30 days (OR 4.96, 95%CI [1.41–17.4]).

**Table 3.** Multivariate Logistic Regression Model of factors associated with self-reported non-adherence (7 day recall)

Variable	OR	[95% CI]		*p*-value
Age (years)	0.97	0.87	1.1	0.60
Female	1.06	0.71	1.58	0.79
Missed clinic appointment in past 6 months	2.23	1.43	3.49	<0.001
Self-efficacy measure: not extremely sure	1.55	1.02	2.34	0.04
Witnessed and experienced household violence in the past year^a^	1.86	1.08	3.21	0.03
Alcohol use in the past month	4.96	1.41	17.44	0.02

^a^Survey question asked: how many times in the past year have you seen an adult in your household physically hurt another person in your home 1+ time versus a. Never.

ALHIV who missed a visit in the past 6 months were more likely to report violence as compared to those who did not miss a visit in the past 6 months (28.8% vs. 10.0%, *p* < 0.0001). But there was no significant association between missing a visit in the past 6 months and alcohol use (1.6% vs. 2.6%, *p* = 0.74).

Age, gender, depression, being bullied for taking medications, and length of time on ART were not found to be associated with non-adherence.

### Self-reported barriers to adherence

As shown in Table 4, the most commonly provided reason for missing medicines was, “forgot”, with over 90% of participants reporting this as a barrier. Other commonly reported reasons included, travel away from home (14%), busy doing other things (11%), and feeling depressed or overwhelmed (6%). Fear of stigma outside the home, wanting to avoid side effects, and not having enough food to eat were also reported as barriers, although less frequently. Most of the assessed barriers were reported by a greater proportion of those that were non-adherent.

**Table 4.** Self-reported barriers to adherence

Barrier	Total *n* (%)	Adherent *n* (%)	Not adherent *n* (%)	*p*-value (Fisher’s)
**Domain: Logistic (rarely, sometimes, often)**	231 (44.5)	79 (21.6)	152 (99.3)	<0.0001
Transportation problems	18 (3.5)	8 (2.2)	10 (6.5)	0.0184
Traveling away from home	71 (13.7)	28 (7.7)	43 (28.1)	<0.0001
Forgot	205 (39.5)	66 (18)	139 (90.8)	<0.0001
Ran out of medicine	19 (3.7)	6 (1.6)	13 (8.5)	0.0004
Busy doing other things	55 (10.6)	16 (4.4)	39 (25.5)	<0.0001
Not having enough food to eat	21 (4)	8 (2.2)	13 (8.5)	0.0022
Medicine got damaged	4 (0.8)	1 (0.3)	3 (2)	0.0790
**Domain: Regimen (rarely, sometimes, often)**	18 (3.5)	5 (1.4)	13 (8.5)	0.0002
Experienced side effects	18 (3.5)	5 (1.4)	13 (8.5)	0.0002
**Domain: Stigma (rarely, sometimes, often)**	35 (6.7)	17 (4.6)	18 (11.8)	0.0062
Fear of stigma by people outside your home	26 (5)	12 (3.3)	14 (9.2)	0.0077
Fear of stigma by people inside your home	16 (3.1)	8 (2.2)	8 (5.2)	0.0918
**Domain: Knowledge/Beliefs (rarely, sometimes, often)**	23 (4.4)	9 (2.5)	14 (9.2)	0.0016
Religious belief – Yes/No	2 (0.4)	1 (0.3)	1 (0.7)	0.5031
Not fully understanding why you were taking the medicine	11 (2.1)	3 (0.8)	8 (5.2)	0.0035
Felt the medicines would be harmful to you	8 (1.5)	6 (1.6)	2 (1.3)	1.0
Did not think the medicines would really work	8 (1.5)	2 (0.5)	6 (3.9)	0.0098
**Domain: Child (rarely, sometimes, often)**	18 (3.5)	6 (1.6)	12 (7.8)	0.0010
Too sick to collect the medicines	10 (1.9)	4 (1.1)	6 (3.9)	0.0714
Tired of having to take the medicines	8 (1.5)	2 (0.5)	6 (3.9)	0.0098
**Domain: Emotional (rarely, sometimes, often)**	32 (6.2)	12 (3.3)	20 (13.1)	<0.0001
Felt depressed or overwhelmed	32 (6.2)	12 (3.3)	20 (13.1)	<0.0001
**Other Barriers (rarely, sometimes, often)**	1 (0.2)	0 (0)	1 (0.7)	0.2948

## Discussion

To our knowledge, this is the first published study in the southern African region to demonstrate that treatment self-efficacy, alcohol use, and violence may be associated with adherence amongst ALHIV. It is also the first study to examine adherence amongst ALHIV in Malawi. We found that self-reported adherence was poor with nearly half (45%) of all ALHIV in this setting reporting missing ART in the past month. The most commonly reported barriers to adherence included forgetting (>90%), travel from home (14%), and busy doing other things (11%). Drinking alcohol in the past month (OR 4.96 95% [1.41–17.4]), witnessing or experiencing violence in the home in the past year, and poor treatment self-efficacy were each found to be independently associated with missing ART in the past week. Strengths of the study include a representative sample of adolescents at all stages in their ART treatment course-those new to treatment as well as those established on ART, including those on second line. Furthermore, the surveys were administered anonymously perhaps decreasing social desirability bias and facilitating more accurate self-reporting of adherence.

Reported adherence in our study is similar to the overall level found in a recent systematic review of adherence to ART amongst ALHIV (62.3% [CI: 57.1–67.6]), but worse than the level found for Africa (83.8% [CI: 8.9–88.7]) in the same review [5]. One reason for this may be that South Africa was over-represented in the review. Studies from other countries in Africa have reported similar levels of self-reported adherence found in our study. Studies examining adherence amongst adolescents in Nigeria and Cote d’Ivoire found that only 59.2% and 67%, respectively, had self-reported 100% adherence [10,38]. The risk of drug resistance with poor adherence is a significant concern, particularly given the limited ART options available in settings like Malawi.

As reported in several studies, the most common self-reported barrier to adherence was simply forgetting to take ART [10,14,23], followed by travel away from home and being busy. Based on these findings, future interventions may want to help equip adolescents and their families with improved planning and problem-solving skills. For example, development of treatment schedules linked to realistic daily activities, and perhaps medication reminder systems [39,40] as well as strategies to maintain adherence while traveling. In addition, exploration of these commonly reported barriers such as “forgot” and “travel” might be helpful in teasing out underlying challenges. When this study was conducted, the recommended first-line regimen in Malawi was a fixed-dose combination of zidovudine, lamivudine, and nevirapine taken twice daily. Long-acting or once daily fixed-dose formulations may help to address the most commonly reported barrier in this study – namely forgetting [41,42].

Substance abuse and violence in the home has previously been reported to be associated with worse adherence [21,43]. In our study, use of alcohol in the past month was highly correlated with non-adherence (OR 4.96, 95% CI [1.41–17.4]). To our knowledge, this association has not yet been documented amongst ALHIV in Southern Africa. Alcohol use typically emerges in adolescence, and in most African countries, access to alcohol is unregulated [44,45]. The relationship between substance use and non-adherence in the African setting deserves further exploration.

Violence during adolescence is associated with a higher risk of adverse health outcomes and mortality [21]. In a recent analysis of the Pediatric HIV/AIDS Adolescent Cohort (8–15 years old) [21], youth who reported exposure to violence in the past year were more likely to also report non-adherence (44% vs. 23%, *p* < 0.001) and were more likely to have unsuppressed viral loads (AOR (95% CI), 1.91 (1.02, 3.55)). This data closely resembles our own results that demonstrate that exposure to violence is independently associated with self-reported non adherence (AOR (95% CI), 1.86 (1.08, 3.22)). There are several potential reasons for this association. Violence may be a proxy indicator for a distressed household, where supervised medication administration is not a priority. However, other potential proxy indicators such as change in caregiver, or experience of household deaths were not found to be significantly associated with non-adherence. Violence experienced by the adolescents themselves may also be an expression of HIV-related stigma and can lead to depression, which can lower adherence levels [46]. Indeed, both being bullied for taking medication and depression were found to be significantly associated with adherence in the sex/age adjusted univariate analysis, however, they did not remain significant in the multivariable logistic regression. Another explanation is that exposure to violence leads to emotional trauma that may independently affect one’s ability to adhere to ART. Studies that can formally explore mechanisms of the violence–adherence relationship may be helpful to shape interventions.

In combination, these results suggest that psychosocial support services for ALHIV are critical to optimize treatment adherence and overall health outcomes. Addressing these challenges will likely require a multi-faceted approach at the patient-level, healthcare provider level, and healthcare systems level. Examples of health services that might be helpful include – availability of once daily fixed-dose combination regimens [41]; addressing psychosocial contexts by screening for substance abuse, depression, bullying and violence during routine ART refill visits [4] and providing training and support for development of staff to provide adolescent-focused psychosocial counselling [47–49]; development of adolescent friendly counselling and support services via the formation of adherence-support clubs [50].

To our knowledge, our study is the first published study to examine the relationship between treatment self-efficacy and ART adherence amongst ALHIV in sub Saharan Africa. Evidence from Thailand and USA demonstrates that treatment self-efficacy positively correlates with ART adherence in adolescents [51,52]. In our study, treatment self-efficacy was independently associated with ART adherence. Inclusion of a brief self-efficacy measure during routine ART clinic visits may help to identify adolescents at risk of poor adherence, and interventions aimed at improving self-efficacy may improve adherence in this population. Further, an important and easy to assess factor associated with non-adherence was missed clinic visit in the past 6 months. In addition, ALHIV who missed a visit in the past 6 months were more likely to report violence. If ALHIV are identified as having missed a clinic visit, further inquiry into reasons and addressing identified barriers may help prevent poor adherence outcomes.

This study does have important limitations. The study is cross-sectional, and stronger conclusions would be possible with a longitudinal cohort. We did not examine protective factors such as resilience. Additional research examining protective factors such as resilience would further expand our understanding of adherence amongst ALHIV. We cannot definitively conclude that our findings are representative of all HIV-positive adolescents in Malawi. The study sites include patients from a wide geographic area and function mainly as primary ART clinics; however, they are also referral centres for ALHIV and therefore cases seen here may be more complicated than elsewhere in Malawi. Another potential limitation of this study is that adherence was assessed only through adolescent self-report. Viral load data would have been very helpful in assessing the clinical relevance of self-reported adherence; however, monitoring of viral load was not routinely in place. However, a recent meta-analysis of over 50 studies from 53 countries found that the prevalence of adherence when using viral suppression was comparable to adherence estimated by self-report 59.1% (95% CI 51.8–66.4%). Moreover, self-reporting of medication adherence by adolescents has been found to be relatively accurate and rates of non-adherence reported in studies using laboratory assays are consistent with rates reported in studies using self-report [23,52–55]. A major challenge with self-report is potential overestimation of adherence due to social desirability bias, however, in our study self-reporting was done anonymously thus potentially reducing bias.

## Conclusions

In summary, there is a pressing need for better interventions to assist ALHIV to remain adherent to ART. Since the completion of this study, the available ART regimens in Malawi have remained relatively unchanged, with few new service modifications that specifically address adolescent ART adherence issues. Our findings are, therefore, not only still relevant but highlight important considerations for optimizing treatment adherence for ALHIV in 2017. We found a very high rate of self-reported non-adherence. We also identified several important modifiable associations with ART non-adherence such as alcohol use, violence in the home, and low treatment self-efficacy that still remain largely unexamined in Southern Africa. Programmes specifically tailored to address those challenges most pertinent to ALHIV may help improve adherence to ART.
